# The Problem of Incipient Insanity

**Published:** 1914-03-07

**Authors:** 


### THE FUTURE OF MENTAL HOSPITALS.

### The Problem of Incipient Insanity.

The position of the medical man in general practice who has to deal with a patient exhibiting signs of early mental disorder remains very unsatisfactory, for, just at the very time when it would be to the advantage of the individual concerned to have his life regulated on certain lines, whether he be agreeable or not, the doctor finds himself hampered by legal disabilities, unless he resorts to certification. As the law stands at present, any person who receives into his house for payment a patient whose symptoms, even for a short time, may reasonably bring him within the category of the insane, is liable to be convicted of a misdemeanour. Consequently, in many instances where it is practically certain that a few months' rest with plenty of fresh air and nourishment will carry the patient through a mental breakdown, the physician in attendance feels compelled to advise certification. In the case of the well-to-do this difficulty is to some extent minimised, as arrangements can be made for placing the invalid in the care of an alienist who devotes his time to this work. But, on the other hand, residence in a doctor's house is far too expensive for persons of small means; because successful treatment of mental illness invariably necessitates a long convalescence, and even small charges represent a heavy outlay when it is a matter of eight months' or a year's stay. In regard to incipient insanity the great need of the layman is that he may have the same opportunity of being cured of a threatening mental illness as he has in regard to diseases of the heart, lungs, stomach, or other organs; and of the practitioner that he may give his patients the full benefits of modern scientific measures which experience shows will restore health to the disordered brain if brought into play soon enough. Under the present Lunacy Laws the practitioner often finds it difficult to carry out such treatment without placing himself or the patient's friends in an illegal position unless he resorts to certification from the outset. It is quite clear to all who have much to do with practical medico-psychology that the interests of the public in connection with the development of treatment must suffer, and the efficiency of mental hospitals be impaired, unless something is done to give responsible medical men more freedom in dealing with cases of incipient insanity.

The necessity for fresh legislation in regard to the care of so-called borderland cases was emphasised by the Medico-Legal Society recently when it passed a resolution to the effect that, in the opinion of the members then met together, the early treatment of incipient insanity without certification has important advantages. But, whilst alienists are agreed that it is in the best interests of patients exhibiting early signs of mental breakdown that they should come under systematic institutional treatment as soon as possible, the specialist has the same difficulties as the general practitioner in that, with one exception, he cannot obtain these advantages for an ordinary well-to-do patient without placing him under certification of lunacy. The exception is, of course, that arrangements may be made for borderland cases to enter certain registered institutions of the insane as "voluntary boarders," and that after so many weeks' or months' submission to the routine treatment therein carried out, such patients can, when restored to health, return to their ordinary occupations without having incurred the stigma of certification, or acquired the detested name of lunatic—at any rate from the official point of view. Unfortunately, compared to the number of ordinary asylums, mental hospitals wherein voluntary boarders can be received are comparatively few.

What, then, is to be the future of such institutions as the proposed hospital for mental diseases which is about to be founded in London through the munificence of Dr. Henry Maudsley? Clearly, under existing laws patients suffering from incipient insanity will no more be able to be received therein than in the homes of doctors or private persons. On the other hand, if everyone who exhibits serious mental symptoms is to be certified before admission to such a hospital, the latter will at once have the same status as an asylum, and the very object for which institutions of this kind should be founded—namely, the relief of mental disorder before it has thoroughly incapacitated its victims and brought them within the category of the insane—must obviously be defeated.

### Definition of "Incipient."

There is, of course, another aspect of the question, as was clearly pointed out by Dr. C. H. Bond, one of His Majesty's Commissioners in Lunacy, at the aforementioned meeting, when he drew attention to the great care needed to prevent the abuse of any slackening of present regulations, so that the unfortunate victims of mental disease may continue to be thoroughly protected against unscrupulous persons inclined to profit by their trouble. Possibly the suggestion that the term "incipient" should be clearly defined so as to indicate either the first attack of mental illness, or some period of time from the onset thereof, will be acted upon in future legislation, so that it shall be understood during what period a medical man may be able to treat without certification cases with severe symptoms. It must be remembered that at the present time, under certain circumstances, it is possible to secure treatment without certification for a month, it being, however, necessary for this purpose that the patient be admitted to the wards of a Poor-Law infirmary. This is freely done in the case of poor persons, and it is, moreover, found that a considerable percentage of such invalids so far improve as to be no longer certifiable within the prescribed period of twenty-eight to thirty-one days allowed by the Lunacy Act—a fact which obviously supports the resolution referred to above.

